# Genome-wide analysis of *OFP* gene family in pepper (*Capsicum annuum* L.)

**DOI:** 10.3389/fgene.2022.941954

**Published:** 2022-09-30

**Authors:** Yin Luo, Shimei Yang, Xirong Luo, Jing Li, Tangyan Li, Xiangqun Tang, Feng Liu, Xuexiao Zou, Cheng Qin

**Affiliations:** ^1^ Longping Branch, College of Biology, Hunan University, Changsha, China; ^2^ Engineering Research Center of Zunyi Pepper Germplasm Resources Conservation and Breeding Cultivation of Guizhou Province, Department of Modern Agriculture, Zunyi Vocational and Technical College, Zunyi, China; ^3^ Key Lab of Zunyi Crop Gene Resource and Germplasm Innovation, Zunyi Academy of Agricultural Sciences, Zunyi, China; ^4^ College of Horticulture, Hunan Agricultural University, Changsha, China

**Keywords:** pepper, *OFP* genes, phylogenetic tree, expression characteristics analysis, qRT-PCR

## Abstract

Ovate family proteins (OFPs) are transcriptional inhibitors that regulate plant growth and development and play important roles in the synthesis of secondary cell walls during pollen development. This study identified the pepper *OFP* gene family based on the genome-wide analysis and used bioinformatics methods to provide a fundamental profile of the gene family. 74 *OFP* genes with typical Ovate domain were identified in cultivated pepper Zunla-1, wild pepper Chiltepin and CM334. Chromosome mapping revealed that *CazOFP* genes were unevenly distributed on 11 chromosomes and Chr00 in Zunla-1, *CacOFP* genes on 12 chromosomes in Chiltepin, and *CamOFP* genes on 12 chromosomes and two Scaffflods in CM334. Gene structure analysis revealed that *CaOFP* genes possessed 1-3 exons, and the analysis of physicochemical properties suggested that *CaOFP*s were hydrophilic. Many *cis*-acting elements were identified in the promoter region of *CaOFP* genes, including ABRE, ARE, Box 4, G-box, TC-rich, and TCT-motif. The expression patterns of pepper at different growth stages showed that *CaOFP* genes were actively involved in the growth and fruit development of pepper, and *CazOFP16* and *CazOFP17* were actively involved in response to multiple hormones and stress events. qRT-PCR was also used to verify the expression of *CazOFP* gene in two developmental stages of seven pepper varieties with different fruit shapes, and it was found that *CaOFP* genes may be involved in the formation of fruit type in pepper. This study provides theoretical and practical evidence for future research on the *OFP* gene family.

## Introduction

Transcription factors (TFs) play fundamental roles in the growth and development of higher plants, as well as in their responses to the external environment. Typical TFs in higher plants possess DNA-binding domains, transcription regulation domains, oligomerization sites, and nuclear localization signals; the interaction between these domains and *cis*-acting elements enables TFs to regulate gene expression (Jiang et al., 2011). Ovate family proteins (OFPs) are plant-specific, multigene family members usually with a conserved Ovate domain. Homologs of *OFPs* have been reported in higher plants, mosses, and lycophytes (Wang et al., 2016).

Pepper is an important vegetable with high standards for its quality, spiciness, and shape, but less research has been done to study its fruit shape. Fruit shape is one of the most important quality traits of pepper, and is also one of the main attributes for the classification of pepper fruit types (Paran and Van Der Knaap, 2007). In crops, yield is regulated by environmental factors and fruit morphology. Genes regulating fruit morphology were first identified in tomatoes, and *OFP* genes are highly involved in this process (Liu et al., 2002; Snouffer et al., 2020). According to previous studies, *OFP* genes were found to be involved in the regulation of shape in different varieties of crops, such as rice, tomato, and melon. Moreover, overexpression of *OFP* destroyed plant organ shape in the process of phytohormone regulation, which suggested that *OFP* genes are involved in regulating hormones and signaling pathways within developing organs (Snouffer et al., 2020). *OFP* genes related to pepper fruit shape were cloned from different pepper varieties, and there were significant differences among the different fruit shapes (Tsaballa et al., 2011). A recent study revealed that virus-induced gene silencing of the pepper ortholog *CaOFP20* resulted in increased fruit elongation on two independent backgrounds (Borovsky et al., 2021).

*OFP* genes play important roles in the growth and development of plants, including flowering (Yuan et al., 2016), growth of roots and stems (Xu et al., 2018), and formation of secondary cell walls (Li et al., 2011). Yuan et al. (2016) detected 12 *VvOFP* genes in different tissues, and the expression levels of *VvOFP1*, *VvOFP2*, *VvOFP4*, *VvOFP5*, *VvOFP6*, *VvOFP7*, *VvOFP12*, and *VvOFP17* were relatively high in the week before flowering, suggesting that *OFP* TFs regulate fruit development during anthesis and the week before anthesis, whereas *VvOFP5* and *VvOFP2* are involved in the regulation of flowering and reproductive growth, respectively (Yuan et al., 2016). Li et al. (2011) also found that *AtOFP4* regulates the formation of secondary cell walls *via* its interaction with the KNAX7 protein.

In this study, we systematically characterized the *CaOFP* transcription factors at the genome-wide level, analyzed its phylogenetic tree, physicochemical properties, conserved structure, chromosomal localization, response to biotic and abiotic stresses, and provided a theoretical basis for studying the function of *OFP* genes in pepper.

## Materials and methods

### Material and RNA extraction

Seven pepper varieties with different fruit shapes were used in this study. Plants were grown in the greenhouse of the department of modern agriculture, Zunyi vocational and technical college in the spring of 2022 (Zunyi, Guizhou, 107°045 ′E, 27°710′ N). The pepper fruits were harvested at two different developmental stages including fruit with mature green and fruit at breaker plus 5 days. All experiments contained three biological repeats, and each replicate was a pooled sample of 10 fruits of uniform size from five individual plants. In total, 42 samples were immediately frozen in liquid nitrogen. RNA was extracted from the collected samples using the TianGene RNA Extraction Kit (DP432, Beijing, China). We then added fruit material with a weight of 50–100 ng for aseptic freezing grinding; 450 μL for oscillation mixing. This was transferred to the CS filter column and centrifuged for 3 min (12,000 rpm). The supernatant was transferred from the collection tube with a pipette gun to the Rnase-free centrifuge tube. Then, the supernatant was added and 0.5 times of anhydrous ethanol was mixed into the centrifuge tube and then transferred to the adsorption column CR3 for centrifugation for 30 s (12,000 rpm). A drop of 80 μL DNase I was added to the center of the collecting tube and left at room temperature for 15 min 250 μL of protein-removing solution RW1 was added to the adsorption column CR3, and left to stand at room temperature for 2 min, before being centrifuged for 30 s (12,000 rpm) (this procedure was repeated once). We then took an enzyme-free centrifuge tube and placed the adsorption column in a new centrifuge tube for several minutes (until the rinsing solution RW was dried). 50 μL Rnase-free ddH_2_O was then vertically added to the adsorption column, and the obtained RNA was stored at −80°C for further Quantitative Real-Time Polymerase Chain Reaction (qRT-PCR).

### Identification and characterization of *OFP* TFs in pepper

The whole-genome data (v2.0) of *Capsicum annuum* L. (Zunla-1 and Chiltepin) were downloaded from the database (http://peppersequence.genomics.cn) (Qin et al., 2014). CM334 genome sequence was downloaded from National Center for Biotechnology Information (https://www.ncbi.nlm.nih.gov/, GCA_000512255.2) (S Kim et al., 2014). To identify the *OFP* TFs, the Pfam model (PF04844) was downloaded from the Pfam database (Mistry et al., 2021), and the HMMER package was used for whole genomic and proteomic alignments. The verification was performed using an online tool, the HMMER web server (https://www.ebi.ac.uk/Tools/hmmer/) (Potter et al., 2018). *AtOFPS* were obtained from the *Arabidopsis* reference genome (https://www.arabidopsis.org/).

### Gene structure, conserved motifs, and phylogenetic analysis of *OFP* genes in pepper

The phylogenetic tree was constructed based on the *CaOFPs* protein sequences in pepper and *AtOFPs* protein sequences in *Arabidopsis thaliana*. MEGA X was used to construct a phylogenetic tree of *CaOFP* genes according to the neighbor-joining method with 1000 bootstrap reiterations (Kumar et al., 2016). The structures of *CaOFP* genes were analyzed using the online tool GSDS 2.0 (http://gsds.gao-lab.org/) (Hu et al., 2015), and the analysis of conserved motifs was conducted using Meme (https://meme-suite.org/meme/) (Bailey et al., 2015) and visualized using TBtools (Chen et al., 2020). Physicochemical properties of the *OFP* gene family were analyzed using the online tool Expasy (Artimo et al., 2012).

### Chromosome mapping and collinearity analysis of *OFP* genes in pepper

The structure file of *OFP* genes in pepper was extracted by TBtools to determine their starting and ending position in the chromosomes (Chen et al., 2020). The protein database was constructed using the Zunla-1 genomic protein file, Chiltepin genomic protein file and CM334 genomic protein file for comparison. The collinearity analysis was performed using MCScanX software (Wang et al., 2013). The chromosome mapping and collinearity results of *OFP* genes were visualized using TBtools (Chen et al., 2020).

### Selection pressure and *cis*-acting element analysis of *OFP* genes in pepper

The online tool Pal2nal (http://www.bork.embl.de/pal2nal/) (Suyama et al., 2006) was used for the analysis of selection pressure of nine pairs of paralogous genes. The 2000-bp upstream sequences of the *OFP* genes were extracted using Bedtools (Quinlan, 2014), analyzed using the online tool Plantcare (Lescot et al., 2002), and visualized using TBtools.

### Characterization of *OFP-*gene expression in pepper

Taking zunla-1 as the reference genome, the gene expression data of pepper were obtained from the NCBI (Accession No. GSE45037) and Pepper Information Data Center (Qin et al., 2014; Liu et al., 2017) (http://pepperhub.hzau.edu.cn). Experimental treatment and data analysis were conducted as described by (Liu et al., 2017), and the gene expression data were visualized using TBtools (Chen et al., 2020).

### cDNA synthesis and qRT-PCR analysis

Expression pattern analysis was investigated using qRT-PCR. Its amplification was carried out using SYBR Green Pro Taq HS (Takara, Dalian, China) according to the manufacturer’s instructions. qRT-PCR was performed on an Applied Biosystems 7500 Real-Time PCR System (Applied Biosystems, Foster City, CA, United States) using the following program: 95°C for 30 s, followed by 40 cycles of 95°C for 5 s and 60°C for 30 s. The *CaActin* (GenBank No. DQ832719) and *CaUbiquitin* (GenBank No. AY496112) pepper genes was amplified as two control genes. Three biological replicates and three measurements for each replicate were performed under identical conditions. Analysis of the relative mRNA expression data was performed using 2^*–ΔΔCt*^ (Livak and Schmittgen, 2001). All primers, designed by Primer3plus (http://www.primer3plus.com/cgi-bin/dev/primer3plus.cgi) and used in this study, were listed in Supplementary Table S1.

## Results

### Identification, phylogenetic analysis, and physicochemical properties of pepper *OFP* genes

**FIGURE 1:**
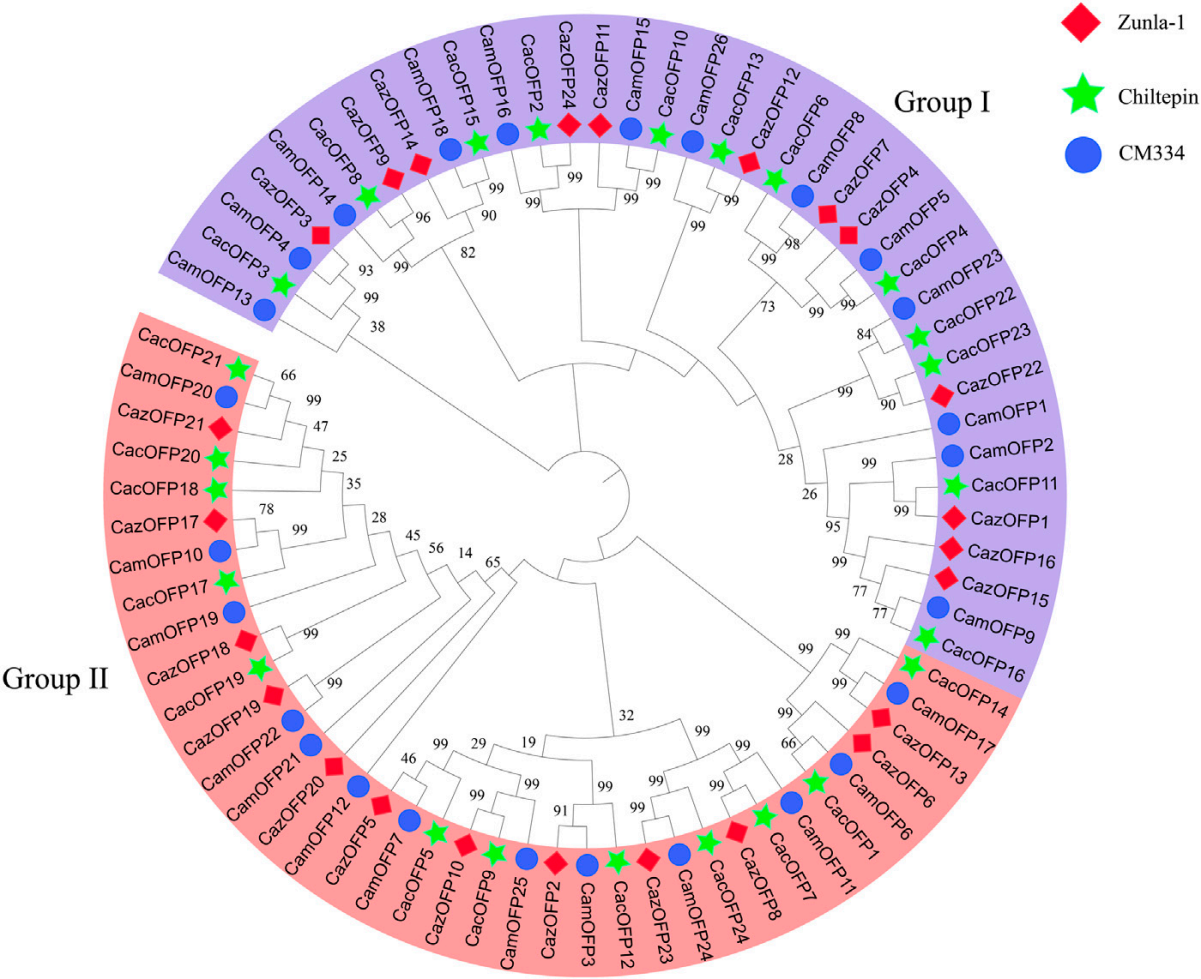
Phylogenetic analysis of *OFP* genes in pepper. Caz: Zunla-1; Cac: Chiltepin; Cam: CM334. The solid diamond with red color represented *CazOFP* gene of Zunla-1; the solid star with green color represented *CacOFP* gene of Chiltepin; and the solid circle with blue color represents *CamOFP* gene of CM334.

After aligning to the pepper proteomic database, 74 *CaOFP* genes were identified in Zunla-1, Chiltepin and CM334 (Table 1; Supplementary Table S2), and the conserved structures were found in all *CaOFP* genes. Phylogenetic data showed that the pepper *OFP* genes were divided into two subfamilies, including Class I and II. The number of *CaOFP* genes of Zunla-1, Chiltepin and CM334 in two subfamilies was 12, 12, and 13, respectively (Figure 1). All *OFP* genes were named according to their positions on the chromosomes. *CaOFPs* consisted of 103–436 amino acids; their molecular mass ranged from 11.82 to 49.45 (kDa), and the isoelectric point (pI) ranged from 4.33 to 9.94. The results indicated that *CaOFPs* are amphoteric proteins, with the instability index ranging from 31.34 to 76.51. The total average hydrophilicity was between -1.030–0.072, and almost all *CaOFPs* were hydrophilic proteins except for *CamOFP25*, which was 0.072 hydrophobic protein.

**TABLE 1:** OFP gene information of pepper.

Gene_id	Rename	Number of amino acids	Molecular weight	pI	Instability index	GRAVY
Capana01g003669	CazOFP1	190	21,309.00	9.54	75.18	−0.502
Capana01g003670	CazOFP2	241	27,214.99	4.33	46.62	−0.509
Capana02g001663	CazOFP3	381	44,895.92	8.40	66.03	−1.020
Capana02g002672	CazOFP4	308	35,371.74	9.64	59.98	−1.009
Capana03g000334	CazOFP5	281	31,625.45	8.66	47.31	−0.691
Capana03g003702	CazOFP6	231	26,239.81	4.67	52.50	−0.630
Capana04g000381	CazOFP7	274	31,578.51	7.09	65.11	−0.717
Capana05g002442	CazOFP8	228	25,629.91	4.68	53.89	−0.358
Capana06g000283	CazOFP9	339	38,208.89	9.66	63.37	−0.797
Capana06g000645	CazOFP10	155	17,809.74	5.38	44.69	−0.048
Capana07g001811	CazOFP11	164	18,936.63	8.99	66.82	−0.678
Capana09g000310	CazOFP12	282	32,488.54	9.14	43.56	−0.873
Capana09g001195	CazOFP13	239	26,380.44	5.37	38.63	−0.308
Capana10g001230	CazOFP14	176	20,071.91	9.11	54.07	−0.520
Capana10g002095	CazOFP15	145	16,616.02	8.67	51.31	−0.385
Capana10g002096	CazOFP16	145	16,616.02	8.67	51.31	−0.385
Capana10g002097	CazOFP17	320	36,192.28	4.55	46.98	−0.531
Capana10g002098	CazOFP18	168	19,101.53	4.42	38.06	−0.330
Capana10g002099	CazOFP19	165	18,469.72	5.20	52.40	−0.420
Capana10g002100	CazOFP20	111	12,768.27	4.66	35.64	−0.372
Capana10g002104	CazOFP21	320	36,161.30	4.83	46.56	−0.529
Capana11g000348	CazOFP22	135	15,068.29	7.64	48.28	−0.591
Capana12g002165	CazOFP23	250	28,182.00	4.35	55.62	−0.657
Capana00g002905	CazOFP24	406	46,225.56	9.72	51.58	−0.823
Capang01g001844	CacOFP1	231	26,215.81	4.62	51.89	−0.594
Capang01g003395	CacOFP2	406	46,197.50	9.68	51.17	−0.821
Capang02g001492	CacOFP3	381	44,981.97	8.21	67.09	−1.030
Capang02g002400	CacOFP4	335	38,506.07	8.92	67.33	−0.986
Capang03g000309	CacOFP5	281	31,548.33	8.67	47.72	−0.707
Capang04g000354	CacOFP6	264	30,636.70	8.75	68.19	−0.672
Capang05g001990	CacOFP7	227	25,532.79	4.68	53.23	−0.353
Capang06g000272	CacOFP8	339	38,208.89	9.66	63.37	−0.797
Capang06g000600	CacOFP9	155	17,809.74	5.38	44.69	−0.048
Capang07g001809	CacOFP10	164	18,936.63	8.99	66.82	−0.678
Capang08g000595	CacOFP11	192	21,483.16	9.54	76.51	−0.505
Capang08g000596	CacOFP12	274	30,519.62	4.33	43.22	−0.407
Capang09g000292	CacOFP13	282	32,520.60	9.14	43.56	−0.881
Capang09g000909	CacOFP14	239	26,394.46	5.37	37.50	−0.308
Capang10g001043	CacOFP15	340	38,301.09	9.75	68.07	−0.807
Capang10g001855	CacOFP16	147	16,850.28	8.68	50.75	−0.366
Capang10g001856	CacOFP17	320	36,274.43	4.55	44.01	−0.515
Capang10g001857	CacOFP18	315	35,270.45	4.94	33.27	−0.520
Capang10g001859	CacOFP19	238	27,195.28	4.77	44.44	−0.541
Capang10g001861	CacOFP20	313	35,222.48	5.25	43.47	−0.406
Capang10g001863	CacOFP21	320	36,140.41	4.97	46.63	−0.509
Capang11g000323	CacOFP22	128	14,703.09	8.77	45.06	−0.669
Capang11g000324	CacOFP23	120	13,854.05	8.25	45.97	−0.594
Capang12g001856	CacOFP24	250	28,210.05	4.39	54.54	−0.661
PHT93985.1	CamOFP1	368	40,626.74	4.91	46.10	−0.635
PHT95520.1	CamOFP2	126	14,074.08	6.40	63.68	−0.112
PHT95521.1	CamOFP3	304	33,830.27	4.40	41.39	−0.427
PHT90291.1	CamOFP4	421	49,484.22	8.93	63.72	−1.017
PHT91194.1	CamOFP5	335	38,478.01	8.92	67.91	−0.985
PHT85943.1	CamOFP6	231	26,242.83	4.62	53.79	−0.605
PHT88768.1	CamOFP7	281	31,602.46	8.67	45.94	−0.682
PHT84793.1	CamOFP8	436	49,428.19	5.99	55.79	−0.356
PHT82131.1	CamOFP9	145	16,616.02	8.67	51.31	−0.385
PHT82132.1	CamOFP10	320	36,192.28	4.55	46.98	−0.531
PHT82493.1	CamOFP11	226	25,444.77	4.68	54.56	−0.318
PHT79195.1	CamOFP12	103	11,817.49	4.59	35.57	−0.359
PHT79899.1	CamOFP13	130	15,152.68	8.22	31.34	−0.165
PHT80370.1	CamOFP14	339	38,294.99	9.70	62.54	−0.810
PHT76741.1	CamOFP15	164	18,936.63	8.99	66.82	−0.678
PHT74641.1	CamOFP16	337	38,375.76	9.62	51.84	−0.859
PHT72525.1	CamOFP17	239	26,380.44	5.37	38.63	−0.308
PHT70189.1	CamOFP18	342	38,563.50	9.94	66.25	−0.833
PHT71118.1	CamOFP19	190	20,883.52	4.63	39.97	−0.253
PHT71146.1	CamOFP20	320	36,161.30	4.83	46.56	−0.529
PHT71148.1	CamOFP21	111	12,768.27	4.66	35.64	−0.372
PHT71152.1	CamOFP22	165	18,376.64	5.35	50.32	−0.408
PHT69076.1	CamOFP23	135	15,353.64	8.61	47.65	−0.748
PHT65371.1	CamOFP24	254	28,626.59	4.43	55.85	−0.671
PHT62009.1	CamOFP25	120	13,567.65	4.36	50.43	0.072
PHT60812.1	CamOFP26	129	14,752.58	4.54	35.48	−0.457

### Structure characterization and motif analysis of *CaOFP* genes

**FIGURE 2:**
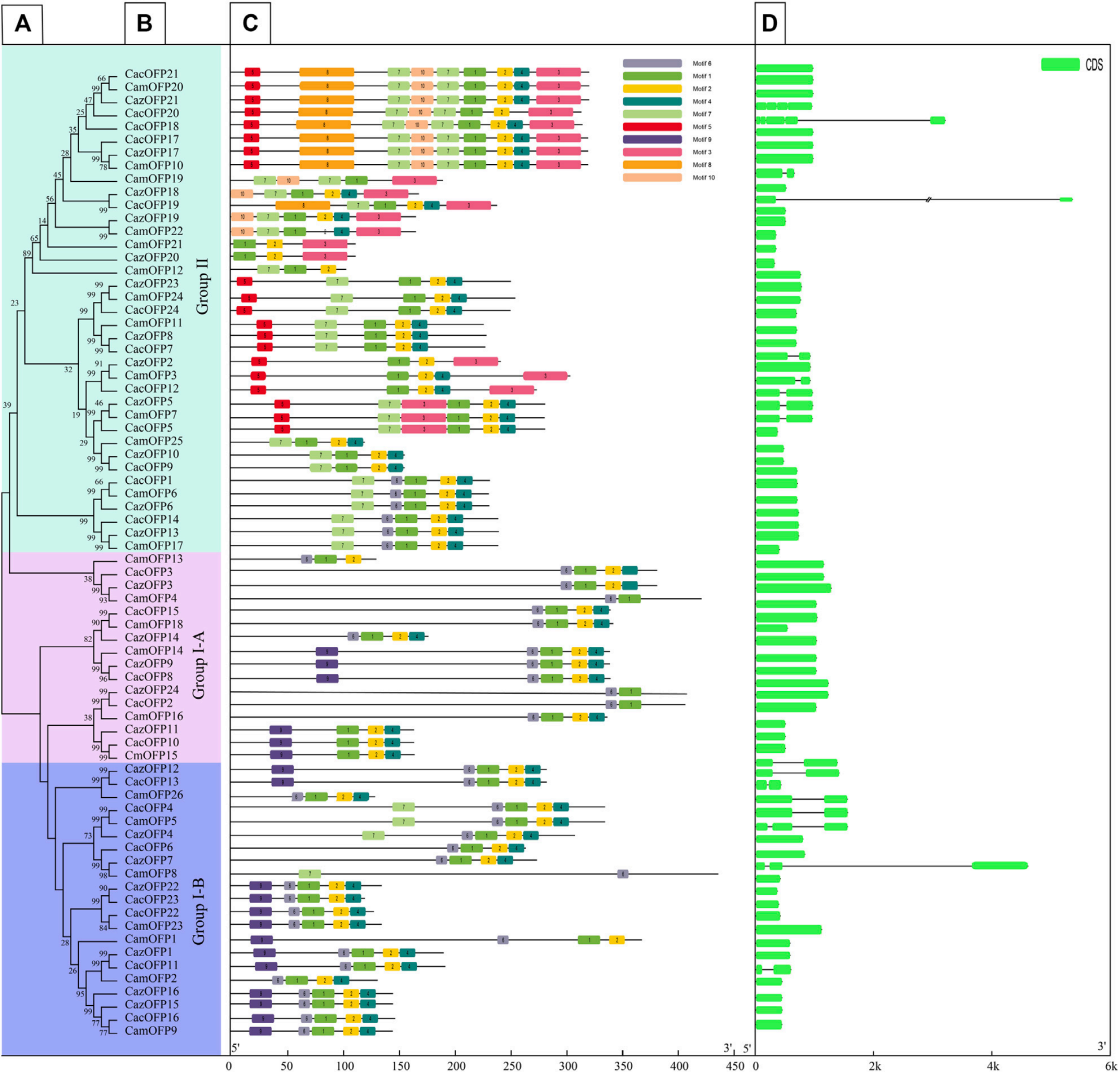
Characteristics analysis of all OFP gene family in pepper. [**(A**,**B)** Phylogenetic tree analysis, **(C)** Gene motifs, **(D)** Gene structure].

**FIGURE 3:**
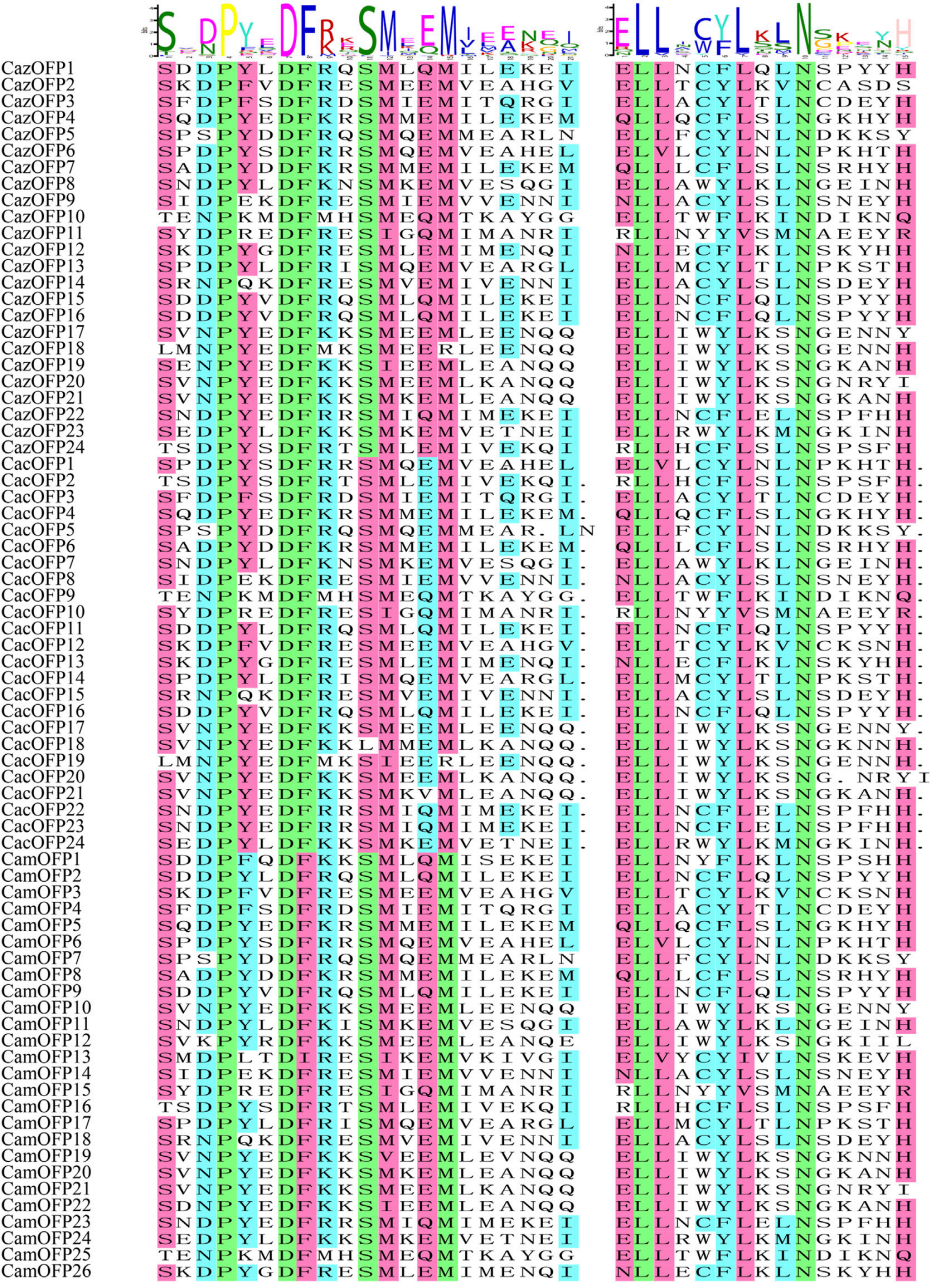
Sequence alignment of OFP transcription factor motifs in pepper.

The motif analysis of *OFP* genes based on 10 motifs showed that the highest number of motifs was recorded for *CazOFP17*, *CazOFP21*, *CacOFP17*, *CacOFP18*, *CacOFP20*, *CacOFP21*, *CamOFP10* and *CamOFP20*, containing 9 motifs (Figure 2C). According to their motifs, Class I can be divided into motif 1, motif 2, motif 4, motif 6 and motif 9 as Class I-A subclade and motif 1, motif 2, motif 4, motif 6, motif 7, motif 9 as Class I-B subclade, and *OFP* genes in Group I only contained 4–5 motifs. The 16 *OFP* genes in Class I-A subclade did not contain introns, while the Class I-B subclade had 1-2 exons. The largest number of exons was presented by *CacOFP18*, which contained five exons, and the number of exons in Class II was higher than that in Class I. Sequence comparison of 74 pepper *OFP* genes showed that all *OFP* genes contained motif1 and 69 *OFP* genes contained motif2 with the typical conserved structure of OVATE domain except for *CazOFP24*, *CacOFP2*, *CamOFP4*, *CamOFP8*, and *CamOFP19* (Figure 3). Gene structure analysis showed that the *OFP* genes possessed 1-5 exons (Figure 2D).

### Chromosome mapping and collinearity analysis of *CaOFP* genes

**FIGURE 4:**
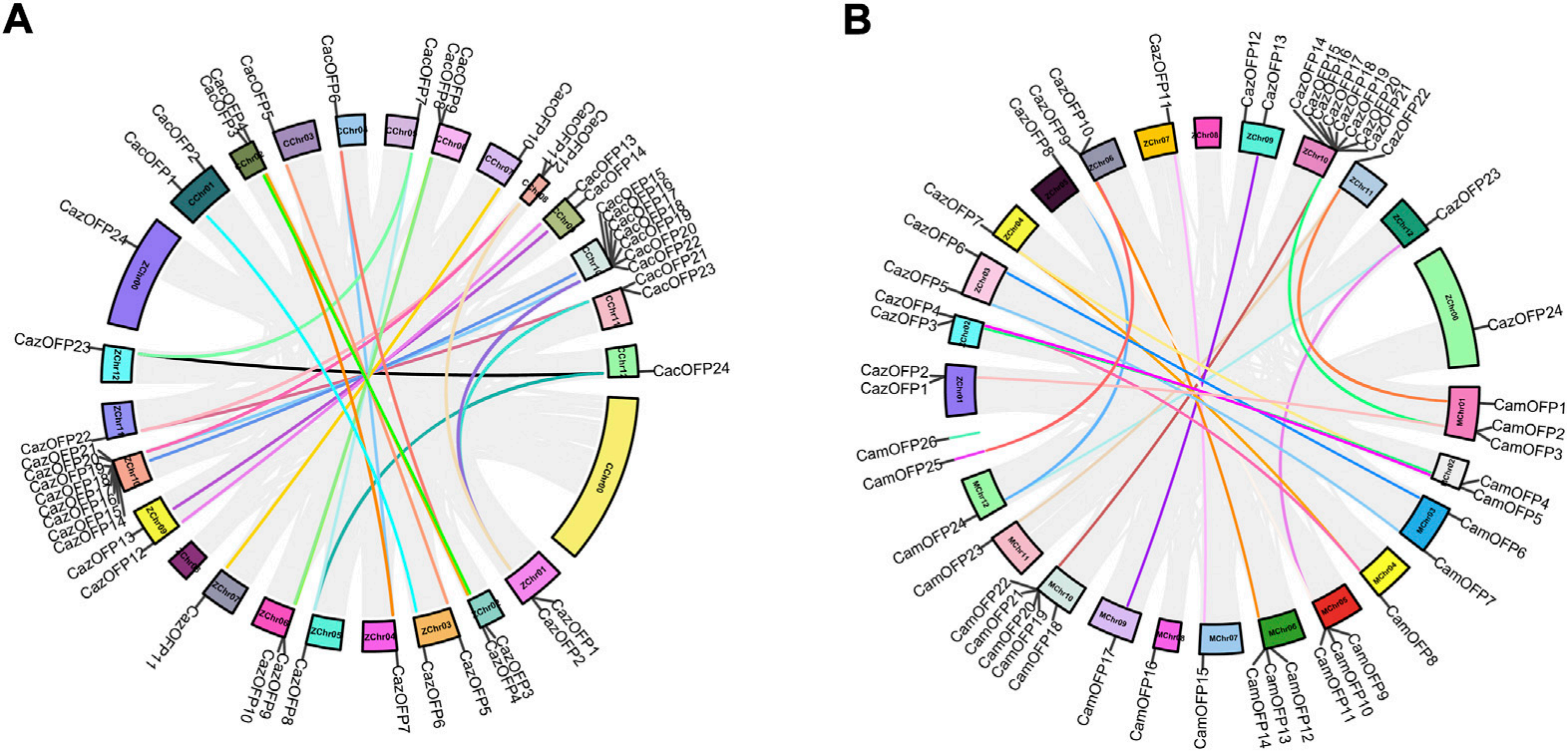
Chromosomal localization and collinearity analysis of *OFP* genes in pepper. [**(A)** ZChr: Zunla-1; CChr: Chiltepin. **(B)** ZChr: Zunla-1; MChr: CM334].

Chromosome mapping analysis showed that 24 *CazOFP* genes were unevenly distributed on 11 chromosomes and Chr00 in Zunla-1, and 24 *CacOFP* genes unevenly on 12 chromosomes in Chiltepin (Figure 4A). Among them, ZChr10 and CChr10 had the highest number of *OFP* genes (8 and 7 genes, respectively), while Chr04, Chr05, Chr07, and Chr012 in Zunla-1 and Chiltepin had only one *OFP* gene each. Interestingly, only ZChr00 contained one *OFP* gene Six genes (*CaOFP15*–*CaOFP21*) on ZChr10 and CChr10 were tandem repeats with colinear regions as well as other chromosomes in Zunla-1 and Chiltepin, but ZChr08 vs. CChr08 and CChr00 vs. ZChr00 did not have colinear regions (Figure 4A). In another comparison (Figure 4B), most chromosomes had colinear regions except for ZChr00, ZChr08, MChr08, and scaffold2890 in Zunla-1 or CM334.

### Analysis of evolutionary selection pressure of *CaOFPs*

Gene synonymous substitution rate (*K*_s_), non-synonymous substitution rate (*K*_a_), and their ratio (*K*_a_/*K*_s_) can reflect the selection pressure in the evolutionary process. The evolutionary selection pressure (*K*_*a*_ */K*_*s*_) of the seven pairs of paralogous genes identified among 74 *CaOFP* genes were greater than one, suggesting that *OFP* genes were subjected to positive selection. Out of these, nine pairs were under evolutionary selection pressure. The Ka/Ks of two pairs (*CacOFP6*-*CacOFP4* and *CamOFP14-CamOFP18*) were less than one, indicating that they were under purification selection, while their Ka/Ks of the other seven pairs were more than one, while the other seven pairs were all above 1, indicating that these genes were under positive selection during the evolution of the *CaOFP* genes in pepper (Table 2; Supplementary Table S3).

**TABLE 2:** Analysis of the evolutionary pressure of selection on the OFP genes in pepper.

Collateral homologous	Synonymous substitution rate (Ks)	Non-synonymous substitution rate (Ka)	Selective pressure ratio (Ka/Ks)
*CacOFP4-CacOFP8*	2.3995	4.2491	1.7709
*CacOFP6-CacOFP4*	59.1311	3.2503	0.055
*CacOFP16-CacOFP11*	2.1397	2.8974	1.3541
*CacOFP23-CacOFP22*	0.409	0.977	2.389
*CazOFP8-CazOFP23*	2.4928	16.504	6.6207
*CazOFP17-CazOFP21*	1.2441	1.3557	1.0897
*CamOFP11_CamOFP24*	1.1054	6.2469	5.6512
*CamOFP10_CamOFP20*	1.2441	1.3557	1.0897
*CamOFP14_CamOFP18*	2.3596	2.1842	0.9256

### *Cis*-acting element analysis of *CaOFP* genes

**FIGURE 5:**
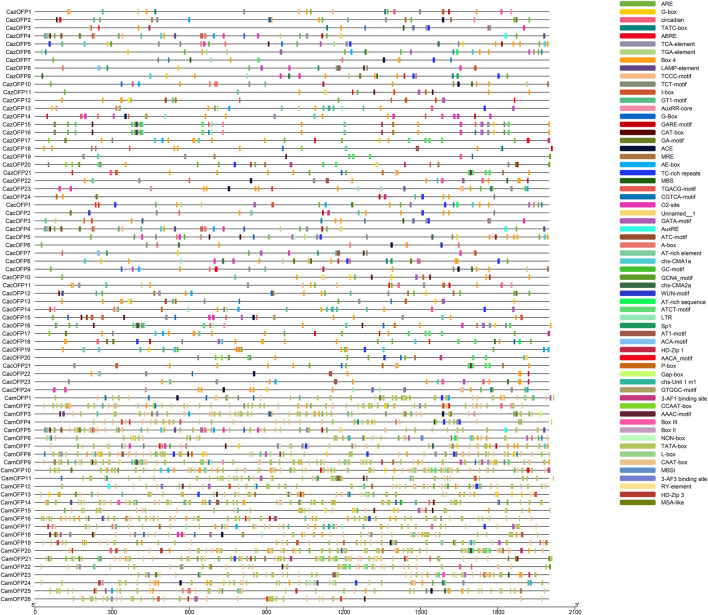
Analysis of *Cis*-acting element in the *OFP* gene family of pepper.

The analysis of the 2000-bp upstream sequence of *CaOFP* genes showed that the *OFP* gene family contained numerous basic elements, such as the CAAT box and TATA box (Figure 5), as well as the ABA-related *cis*-acting element ABRE, anaerobic induction-required *cis*-acting element ARE, photoresponse-related conserved element Box 4, photoresponse-related *cis*-acting element G-box, defense and stress response-related *cis*-acting element TC-rich repeats, photoresponse element TCT-motif, cis-acting element involved in salicylic acid responsiveness TCA-element, MYB binding site involved in drought-inducibility MBS and cis-acting element involved in low-temperature responsiveness LTR (Supplementary Table S4). It was speculated that these genes may respond to biological stress and be involve in plant growth.

### Expression patterns of *OFP* genes in different tissues and development stages of pepper

**FIGURE 6:**
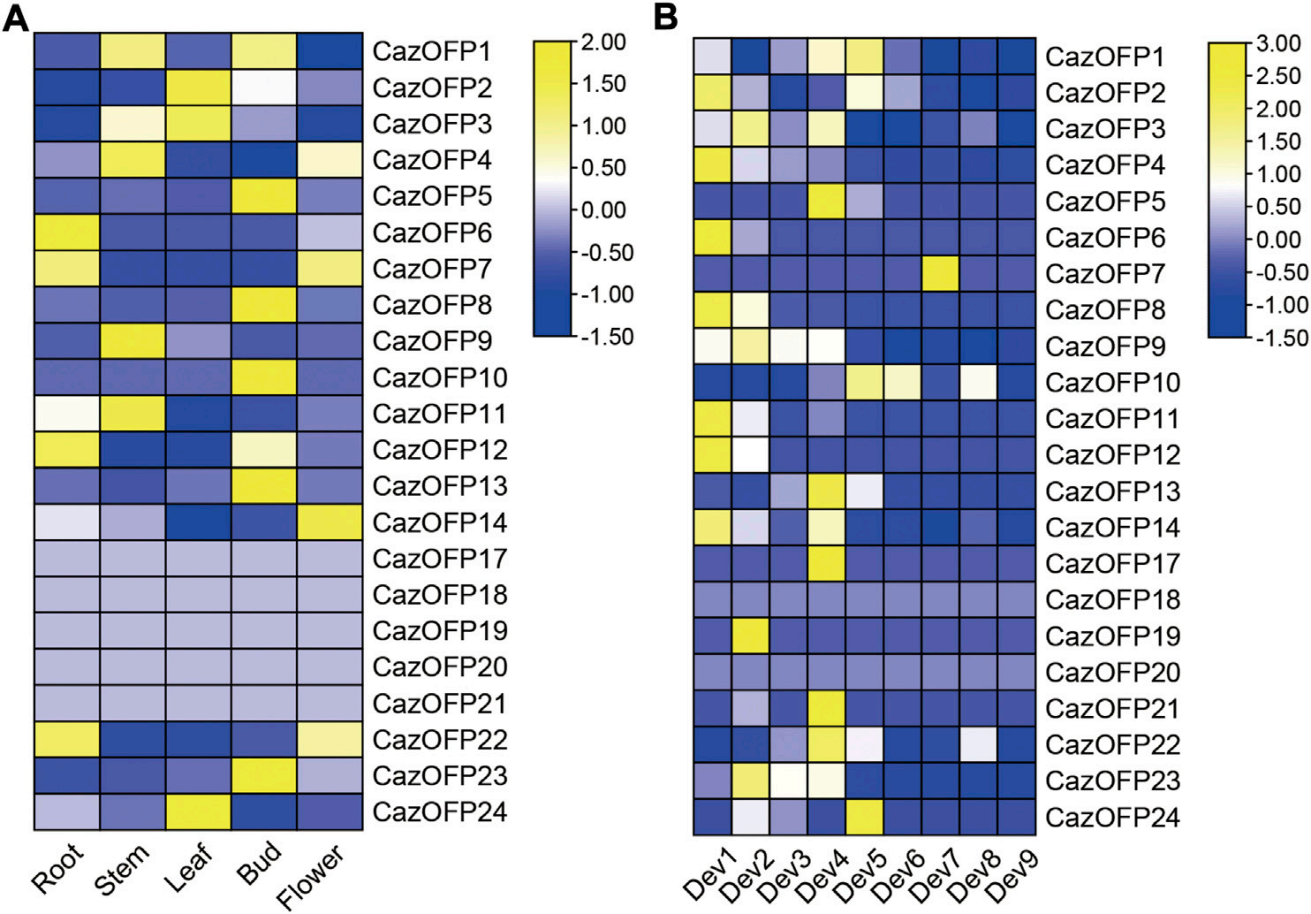
Expression pattern of *OFP* genes in pepper.[**(A)** Roots: developing roots; Stems: developing stems; Leaves: mature leaves; Bud: closed flower buds; Flowers: open flowers. **(B)** Dev1: Fruits with length between 0–1 cm; Dev2: Fruits with length between 1–3 cm; Dev3: Fruits with length between 3–4 cm; Dev4: Fruits with length between 4–5 cm; Dev5: mature green fruit; Dev6: Fruit at breaker (fruit turning red); Dev7: Fruit at breaker plus 3 days; Dev8: Fruit at breaker plus 5 days; Dev9: Fruit at Breaker plus 7 days].

The expression levels of 24 *CazOFP* genes in five tissues including developing roots, developing stems, mature leaves, closed flower buds and open flowers from Zunla-1 pepper data were analyzed. It showed that *CazOFP17*∼*CazOFP21* were not expressed, and other 19 *CazOFP* were expressed in five different tissues on different levels (Figure 6A). During the nine stages of fruit development in Zunla-1, 22 *CazOFP* genes except for *CazOFP18* and *CazOFP20* were expressed in the first eight stages, showing that these *CazOFP* genes were mainly involved in early fruit development. One gene (*CazOFP10*), by contrast, was mainly involved in late fruit development (Figure 6B).

### Characterization of *CazOFP* genes expression patterns under different stresses in pepper

**FIGURE 7:**
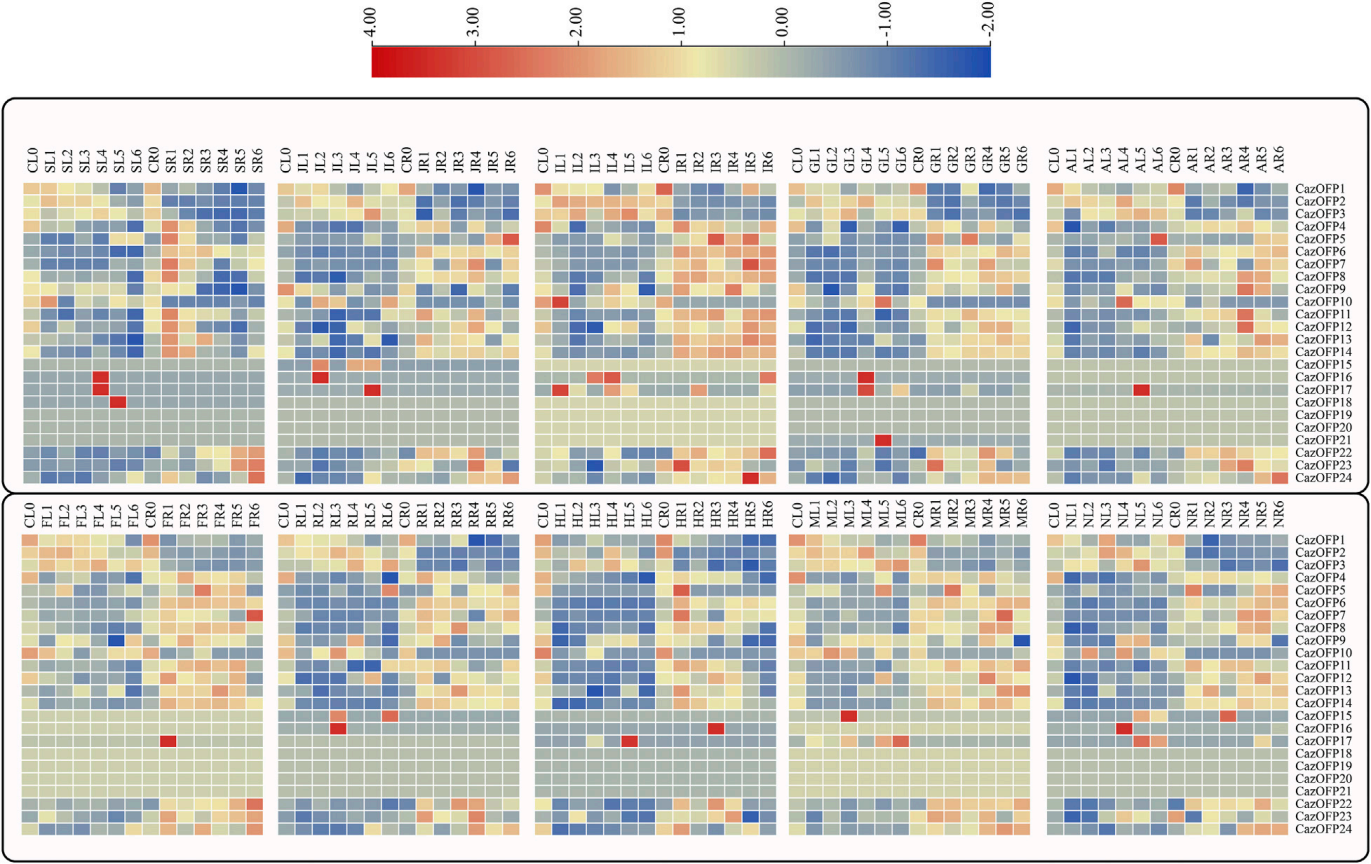
Analysis of *CazOFP* gene expression profiles in pepper. Red: up-regulated expression; Gray: not expressive; Blue: down-regulated expression. CL: Control Leaf; SL: SA Leaf; JL:JA Leaf; IL: IAA Leaf; GL: GA3 Leaf; CR: Control Root; SR:SA Root; JR: JA Root; IR: IAA Root; GR: GA3 Root; AL: ABA Leaf; FL: Cold Leaf; RL: H2O2 Leaf; HL: Heat Leaf; ML: Mannitol Leaf; NL: NaCl Leaf; AR: ABA Root; FR: Cold Root; RR: H2O2 Root; HR: Heat Root; MR: Mannitol Root; NR: NaCl Root.

As showed in Figure 7, the expression of *CazOFP16*, *CazOFP17, and CazOFP18* in pepper leaves was up-regulated upon salicylic acid (SA, 2 mM) treatment. Jasmonic acid (JA, 10 μM) treatment similarly up-regulated the expression of *CazOFP16 and CazOFP17.* Pepper *OFP* genes expression was mainly up-regulated in roots after indole acetic acid (IAA, 2 μM) treatment; the expression of *CazOFP24* in roots was higher than in leaves at a late expression stage. When treated with gibberellic acid (GA_3,_ 2 μM), the expression of *CazOFP16, CazOFP17, and CazOFP21* was higher than that of other *OFP* genes, and the level of up-regulation was greater in roots than in leaves. Abscisic acid (ABA, 30 μM) treatment up-regulated the expression of *CazOFP11 and CazOFP12* in roots at stage AR4, while *CazOFP17* had the highest expression level in leaves at stage AL5. The expression of *CazOFP17* was significantly up-regulated upon salicylic acid (SA, 2 mM), JA, IAA, GA_3_, and ABA treatments, which suggested that *CazOFP17* participates in plant response to the hormone, whereas *CazOFP18, CazOFP19, and CazOFP20* were not expressed.

The expression of *CazOFP17* was up-regulated in roots under cold stress at stage FR1 (Root/Freezing 1), and the expression of *CazOFP16* was up-regulated in leaves at stage RL3 when treated with H_2_O_2_ (Figure 7). Heat stress up-regulated the expression of *CazOFP16* in roots at stage HR3 and that of *CazOFP17* in leaves at stage HL5 (Figure 7). When treated with D-mannitol, the expression of *CazOFP15* was up-regulated in leaves; the expression of *CazOFPs* was higher in roots than in leaves. Salt stress up-regulated the expression of *CazOFP16* in leaves at stage NL4 (Figure 7). In summary, *CazOFP18-21* were not expressed, and the number of genes expressed in roots was higher than that in leaves.

### Expression patterns of *OFP* genes in seven pepper varieties with different fruit shapes by qRT-PCR experiments

**FIGURE 8:**
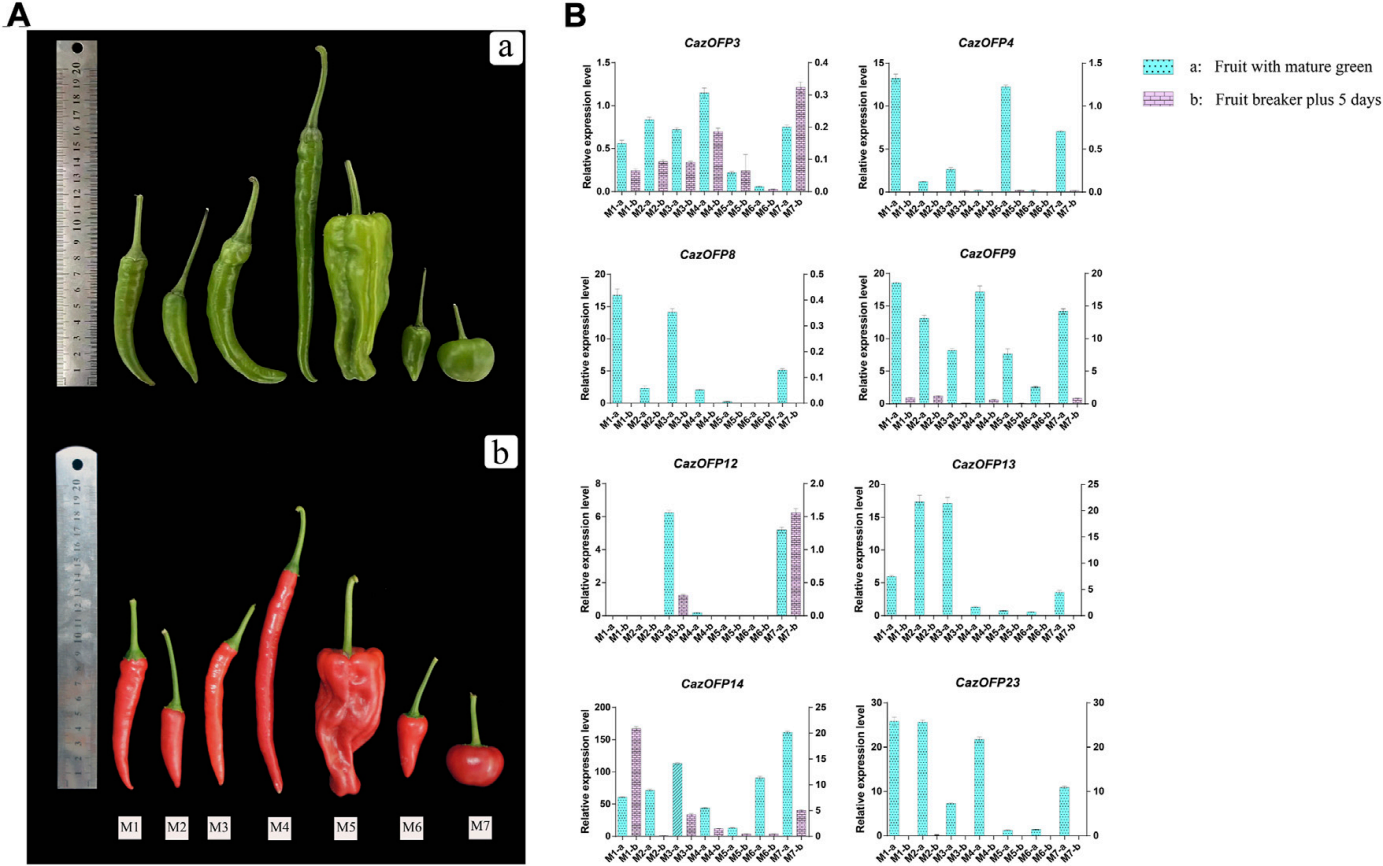
Expression of eight *CazOFP* genes in two developmental stages of seven pepper varieties with different fruit shapes. **(A)** Fruit diagrams of seven varieties with different fruit shapes. These varieties (M1-M7) are zunla-1, ZJ-5, 11C255-1, ZJ11, HYL, ZJ-1 and T803. Above is the fruit development stage 1 (fruit with mature green), Below is the fruit development stage 2 (fruit at breaker plus 5 days). **(B)** The levels of these eight *CazOFP* genes detected by qRT-PCR.

In order to verify wheather *CaOFP* genes involve in pepper development and fruit shape formation (Figure 8A), eight *CazOFP* genes were randomly selected and qRT-PCR performed. These genes except for *CazOFP12* were mainly expressed in developmental stage 1 (fruit with mature green) of seven pepper varieties with different fruit shapes (Figure 8B), indicating that these genes were mainly involved in early fruit development, which were consistent with transcriptome results mentioned above. In seven pepper varieties with different fruit shapes, the expression of *CazOFP3* and *CazOFP12* at developmental stage 2 (fruit at breaker plus 5 days) in T803 was higher than that at developmental stage 1 (fruit with mature green), as well as *CazOFP14* in Zunla-1. Moreover, *CazOFP3*, *CazOFP9* and *CazOFP14* were expressed in two developmental stages of seven pepper varieties, showing that the three genes may be involved in the formation of pepper fruit shape.

## Discussion

### Characteristics of *OFP* gene family in pepper

We identified 24 *CaOFP* genes in cultivated pepper Zunla-1, 24 in wild pepper Chiltepin, and 26 in another cultivated pepper CM334. So far, *OFP* genes have been identified in many species with the development of genome sequencing, such as 18 *AtOFP* genes in *A. thaliana* (Wang et al., 2011), 35 *RsOFP* genes in *Raphanus sativus*, 31 *SlOFP* genes in *Solanum lycopersicum* (Huang et al., 2013), 29 *BrOFP* genes in *Brassica rapa* (Wang et al., 2021), and 8 *VvOFP* genes in *Vitis vinifera* (Wang et al., 2018).

In reported studies, the tomato *SlOFP* genes can be divided into three subfamilies (Huang et al., 2013), and the apple *OFP* genes can be divided into 15 subfamilies by carrying out evolutionary analysis of 727 protein sequences from 32 species (Li et al., 2019), while Liu et al. found that *OFP* genes in angiosperms were divided into 11 subfamilies by phylogenetic tree analysis (Liu et al., 2014). In the study, the *CaOFP* genes were divided into two subfamilies, namely Class I and Class II by identifying and classifying three varieties of pepper (Zunla-1, Chiltepin and CM334). However, these *CaOFP* genes can be subdivided according to their conserved domains and motifs. For example, Class I contains only six motifs, while Class II contains motif1, motif5, motif8, and motif10. These results indicated that the subfamily classification of *OFP* genes was quite different according to their plant properties, conserved domains and exon size. In a previous study, *OFP* genes of *Selaginella moellendorffii*, an early terrestrial plant, were found to have more than dozens of exons (Dangwal and Das, 2018), while there were 1-5 exons in the *CaOFP* gene family of pepper, and Class I-A subclade did not contain introns according to its subgroup classification.

Some studies have found that *OFP* was not only involved in the regulation of plant growth and development, but also in plant stress response and fruit morphology formation. Liu et al. cloned OVATE in tomato for the first time and found that it could control the shape of tomato fruit. A point mutation of OVATE gene led to premature termination of translation, which increased the longitudinal diameter of wild tomato fruit and limited the growth of its neck, thus developing from round fruit to pear fruit (Liu et al., 2002). In the yeast two-hybrid assay, nine OVATE proteins were found to interact with TALE homeobox proteins, and AtOFP1 and AtOFP5 co-regulated the subcellular localization of a TALE homeobox protein BLH1. When these two genes were co-expressed in tobacco leaves, The BLH1 protein was transported from the nucleus to the cytoplasm (Hackbusch et al., 2005). Numerous studies have shown that OFP played a role by interacting with KNOX and BELL transcription factors. For example, Schmitz et al. found that OsOFP2 could regulate KNOX-BELL function to participate in the development process of rice (Schmitz et al., 2015). Another study found that the *CmOFP13* gene *fsqs8.1* could alter the recessive structure of the gene in melon, which could lead to structural variation and affect the melon fruit morphology (Martínez et al., 2022). Current biochemical evidence suggests that OFPs may function in this feedback through interactions with three amino acid loop extension (TALE) proteins, as well as through interactions with additional proteins within signaling pathways (Snouffer et al., 2020). Expression patterns of *OFP* genes in different tissues and development stages of pepper and seven pepper varieties with different fruit shapes indicated that some *CaOFP* genes were actively involved in the growth and development of pepper, and participated in plant stress response process and fruit morphological development through mediating mediates.

### Expression profiles of *CaOFP* genes in pepper

Recent studies have found that *OFP* gene was responsive to salt stress in plants. Tang et al. found that *PpOFP1* had salt tolerance through yeast experiments *in vitro* (Tan et al., 2021). Ma et al. found that the *OsOFP6* overexpression lines had slower water loss and less H_2_O_2_ accumulation under drought condition, while the RNAi lines had faster water loss and higher H_2_O_2_ content, which indicated that *OsOFP6* may have drought resistance in rice (Ma et al., 2017). Characterization of *CaOFP* genes expression patterns under different stresses in pepper showed that *CazOFP15*, *CazOFP16* and *CazOFP17* can respond to regulation and participate in the salt stress process. Aabscisic acid responsiveness, salicylic acid responsiveness, defense and stress responsiveness, drought-inducibility, low-temperature responsiveness were also found in the upstream 2000 bp of pepper *CaOFP* genes. Defense and stress responsiveness, drought-inducibility, low-temperature responsiveness, etc. Now studies on OFP mainly focus on growth and development, including defense and stress response, low temperature response and drought induction, while no other studies under SA, JA, IAA, GA3, ABA, H_2_O_2_, Mannitol stress have been reported. According to the analysis of cis-acting elements in pepper, It is speculated that *CazOFP* plays a functional role in responding to relevant elements, which provides new insights for subsequent studies on pepper’s response to biotic and abiotic stresses. By studying the expression profile of *CazOFP* genes under different tissues and stress conditions, this study provides theoretical support for studying the stress response process of *OFP* and its participation in the regulation of growth and development in pepper.

*OFP*genes have been found to reduce the length of hypocotyls, rosette leaves, stem leaves, inflorescence stems and floral organs in Arabidopsis mutants, while *AtOFP1* regulates the target gene *AtGA20ox1*, resulting in insufficient gibberellin synthesis. These results indicated that OFP transcription factors negatively regulated plant growth and development (Wang et al., 2007). OFP can also promote chlorophyll accumulation and delay leaf senescence. Zhou et al. overexpressed *SlOFP20* in tomato and found that the expressions of genes encoding transcription factors SlGLK1, SlGLK2 and HY5 related to chloroplast development and chlorophyll level were significantly up-regulated, which indicated that OFP had a positive regulatory effect on chlorophyll accumulation and retarding leaf senescence (Zhou et al., 2019). At present, it had been reported that *OFP* was involved in the fruit shape development process of pepper, and CaOVATE was found to be involved in the fruit shape process of tomato through the study of pepper varieties with different fruit shapes (Tsaballa et al., 2011). Expression pattern analysis of *Citrus OFPs* (*CitOFPs*) showed that *CitOFP19* had significantly higher expression level in the ovaries of round pummelo than in those of pear-shaped pummelo (Wu et al., 2022). We also found that the *CazOFP* genes were up-regulated in the early stages of pepper fruit development (Dev1∼Dev5) in the study. However, *CazOFPs* expression tended to be down-regulated in the late fruit stages. The expression levels of *CazOFP9* and *CazOFP13* in thin pepper varieties were higher than those in large pepper varieties, suggesting that *CazOFP9* and *CazOFP13* were involved in the fruit shape development of thin pepper varieties. In brief, *CazOFP* genes with different expression levels in pepper may be involved in plant growth and development, thus playing both positive and negative regulatory roles.

Fruit shape is an important quality and yield trait of pepper. High quality pepper and excellent fruit shape help to improve its market competitiveness and increase economic output value. The mechanism and functional verification of OFP transcription factors regulating the growth and development of pepper will become the focus of future research. With the development of plant genome field, it is helpful to explore more *OFP* gene functions and provide theoretical and practical support for the study of plant shape and morphological development.

## Data Availability

Publicly available datasets were analyzed in this study. The names of the repository/repositories and accession number(s) can be found in the article/Supplementary Material.
